# Isolation of High-Purity Extracellular Vesicles by the Combination of Iodixanol Density Gradient Ultracentrifugation and Bind-Elute Chromatography From Blood Plasma

**DOI:** 10.3389/fphys.2018.01479

**Published:** 2018-10-23

**Authors:** Zsófia Onódi, Csilla Pelyhe, Csilla Terézia Nagy, Gábor B. Brenner, Laura Almási, Ágnes Kittel, Mateja Manček-Keber, Péter Ferdinandy, Edit I. Buzás, Zoltán Giricz

**Affiliations:** ^1^Department of Pharmacology and Pharmacotherapy, Semmelweis University, Budapest, Hungary; ^2^Institute of Experimental Medicine, Hungarian Academy of Sciences, Budapest, Hungary; ^3^Department of Synthetic Biology and Immunology, National Institute of Chemistry, Ljubljana, Slovenia; ^4^EN-FIST Centre of Excellence, Ljubljana, Slovenia; ^5^Pharmahungary Group, Budapest, Hungary; ^6^Department of Genetics, Cell- and Immunobiology, Semmelweis University, Budapest, Hungary; ^7^MTA-SE Immune Proteogenomics Extracellular Vesicle Research Group, Budapest, Hungary

**Keywords:** extracellular vesicles, isolation, exosomes, plasma, iodixanol, density gradient ultracentrifugation, bind-elute chromatography

## Abstract

**Background:** Extracellular vesicles (EVs) (isolated from blood plasma) are currently being extensively researched, both as biomarkers and for their therapeutic possibilities. One challenging aspect to this research is the efficient isolation of high-purity EVs from blood plasma in quantities sufficient for *in vivo* experiments. In accordance with this challenge, the aim of this study was to develop an isolation method in which to separate the majority of EVs from major impurities such as lipoprotein particles and the abundant plasma proteins albumin and fibrinogen.

**Methods:** Samples of rat blood were centrifuged to remove cells, platelets, large EVs and protein aggregates without prior filtration. Density gradient ultracentrifugation was performed by loading plasma sample onto 50, 30, and 10% iodixanol layers and then centrifuged at 120,000 ×*g* for 24 h. Ten fractions (F1-10) were collected from top to bottom. Fractions with the highest EV content were further purified by ultracentrifugation, size exclusion, or bind-elute chromatography. Efficiency and purity were assessed by Western blots. Morphology and size distribution of particles were examined by dynamic light scattering and electron microscopy (EM).

**Results:** The highest band intensities of EV markers Alix, Tsg101 and CD81 were detected by Western blot in F6 of small-scale DGUC (61.5 ± 10.4%; 48.1 ± 5.8%; 41.9 ± 3.8%, respectively) at a density of 1.128–1.174 g/mL, where the presence of vesicles with a mean diameter of 38 ± 2 nm was confirmed by EM and DLS. Only 1.4 ± 0.5% of LDL and chylomicron marker, 3.0 ± 1.3% of HDL marker, and 9.9 ± 0.4% of albumin remained in the EV-rich F6. However, 32.8 ± 1.5% of the total fibrinogen beta was found in this fraction. Second-step purification by UC or SEC did not improve EV separation, while after BEC on HiScreen Capto Core 700 albumin and lipoprotein contamination were below detection limit in EV-rich fractions. However, BEC decreased efficiency of EV isolation, and fibrinogen was still present in EV-rich fractions.

**Conclusion:** This is the first demonstration that DGUC is able to markedly reduce the lipoprotein content of EV isolates while it separates EVs with high efficiency. Moreover, isolation of lipoprotein- and albumin-free EVs from blood plasma can be achieved by DGUC followed by BEC, however, on the expense of reduced EV yield.

## Introduction

Extracellular vesicles (EVs) are released by various cell types and transport different proteins, nucleic acids and lipids generally representing the parental cell (Raposo and Stoorvogel, 2013). EVs have been linked to several physiological or pathological processes as mediators (e.g., immune response, tumorigenesis or cardioprotection; (Giricz et al., 2014; Barile et al., 2017; Sluijter et al., 2018) and they have been shown to have the potential for clinical applications, both as biomarkers or drug delivery systems (D’Souza-Schorey and Clancy, 2012). For example, the role of EVs in the immune system highlights their potential in immunomodulatory therapies, as EVs could be applied in antigen presentation or even as vaccines (Wang et al., 2018). While miRNA and mRNA cargo of EVs allow the use of EVs isolated from blood in cancer diagnostics (Rolfo et al., 2014; Krug et al., 2018). Further research on the composition, *in vivo* functionality, and pharmacological applicability of EVs isolated from blood plasma requires large amounts of intact vesicles purified from other, non-vesicular plasma components, for which suitable isolation methods have not yet been demonstrated.

Currently, the most commonly applied methods for EV isolation from blood plasma are based on differential UC, SEC, filtration, or the combination thereof (Kalra et al., 2013; Boing et al., 2014; Baranyai et al., 2015; Welton et al., 2015; Mol et al., 2017; Sluijter et al., 2018). Isolation of pure EVs from plasma samples faces numerous challenges including aggregation of vesicles (Linares et al., 2015), significant contamination with soluble plasma proteins (Kalra et al., 2013; Welton et al., 2015), and co-isolation with EV-sized lipoproteins (Yuana et al., 2014; Sodar et al., 2016), which could hinder functional and analytical studies on EVs since certain proteins and, particularly lipoproteins, may carry microRNA (Yuana et al., 2014). It was also reported that UC- or SEC-based isolation achieve low recovery of EVs, which may limit their applicability for most analytical and *in vivo* therapeutical goals (Baranyai et al., 2015). Thus, there is a need for more efficient isolation protocols.

Separating particles on the basis of their buoyant densities by DGUC using sucrose or iodixanol has been used for EV isolation from cell culture supernatant and body fluids, most often coupled with other methods such as UC or SEC (Tauro et al., 2012; Iwai et al., 2016). Although, DGUC may result in EVs with less contaminant than that which is obtained by other methods (Tauro et al., 2012; Kalra et al., 2013; Iwai et al., 2016; Konadu et al., 2016; Kowal et al., 2016; Karimi et al., 2018), available isolation strategies involving DGUC have low yield due to the multiple-step protocols (Kalra et al., 2013; Momen-Heravi et al., 2013; Baranyai et al., 2015). In 2018, it was demonstrated that DGUC with iodixanol might successfully separate EVs isolated from blood plasma from similar sized lipoproteins without prior UC; however, low yield and contamination of soluble protein remained significant issues (Karimi et al., 2018). Therefore, further optimization of EV isolation is required.

Recently innovative technologies have emerged for EV isolation. For instance, Corso et al. (2017) reported that by using BEC on Capto Core 700 column cell culture EVs isolated could be separated with high efficiency from protein contaminants. This column does not only exclude EV-sized particles, but also captures negatively charged or hydrophobic proteins and other molecules. Nevertheless, no data has been reported on the applicability of BEC for the isolation of EVs from complex biological fluids such as blood plasma.

Thus, the main objectives of our study were (1) to establish a iodixanol-DGUC protocol for efficient isolation of EVs from blood plasma, (2) to test if BEC could be applied for the efficient isolation of EVs isolated from blood plasma in combination with DGUC, and (3) to assess the yield and separation efficiency of the applied protocols, and (4) to monitor the presence of some of the most abundant soluble protein and lipoprotein contaminations of EV isolates.

## Materials and Methods

For a detailed description of the methods used, please check ‘Supplementary Methods,’ in this paper. Since we did not apply filtering during separation and since the size ranges of exosomes and MVs may overlap, we use the generic term “extracellular vesicles” to refer to the vesicles isolated here, which is in accordance with previous recommendations (Gould and Raposo, 2013).

### Animal Welfare

The investigation conforms to the Guide for the Care and Use of Laboratory Animals published by the US National Institutes of Health (NIH publication No. 85–23, revised 1996), to the EU Directive (2010/63/EU) and was approved by the animal ethics committee of the Semmelweis University, Budapest, Hungary (PE/EA/1784-7/2017).

### Blood Collection and Sample Preparation

**FIGURE 1:**
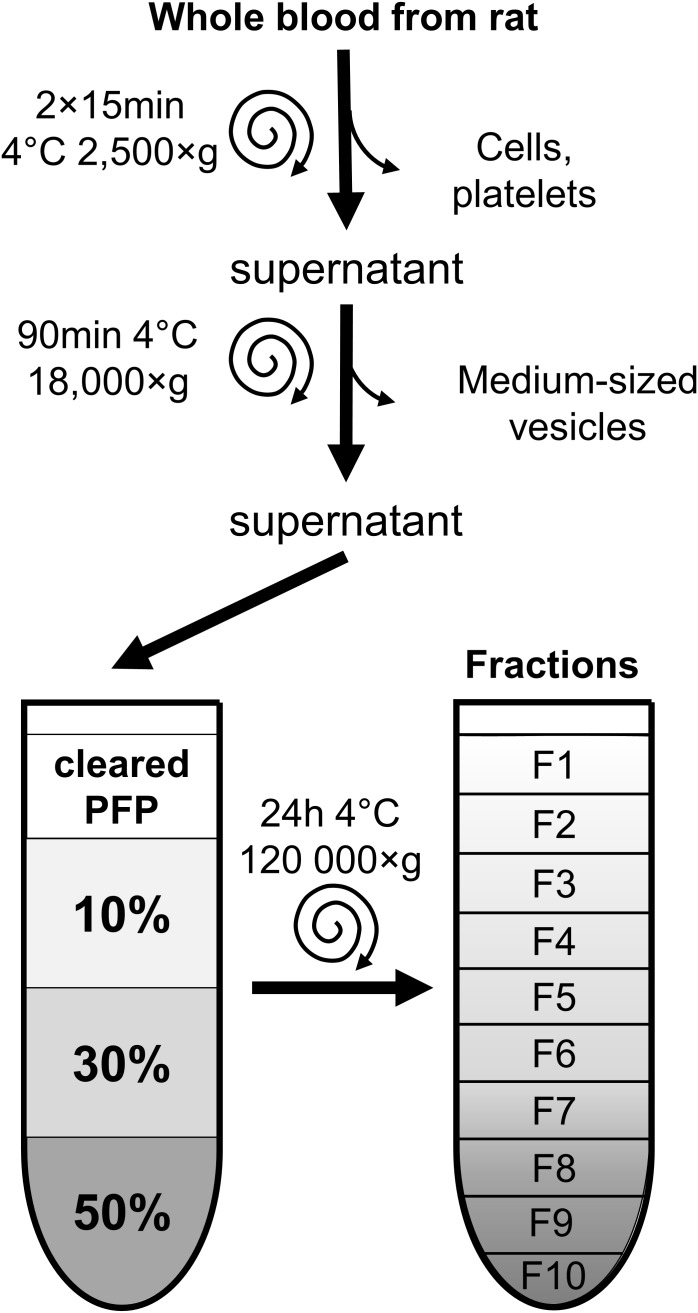
Overview of sample preparation and EV-isolation by iodixanol density gradient ultracentrifugation. Blood samples were centrifuged to remove cells, large and medium-sized particles. The final supernatant platelet free plasma was placed onto 10–30–50% iodixanol gradient layers, then ultracentrifuged for 24 h. Ten fractions were collected from top to bottom and characterized by electron microscopy, Western blot analysis and dynamic light scattering. F, fraction; PFP, platelet-free plasma.

Whole blood was collected from the caudal vena cava of male Wistar rats weighing 200–300 g into Anticoagulant Citrate Dextrose-A vacuum tubes. PFP was obtained by centrifuging samples two times at 2,500 ×*g* at 4°C, for 15 min as previously described (Baranyai et al., 2015). PFP was then centrifuged at 18,000 ×*g* at 4°C, for 90 min, (cleared PFP), then stored at -80°C until isolation procedure (Figure 1). To compare the results of EV isolated from frozen cleared PFP, a parallel study was also carried out on EVs isolated from fresh samples without prior freezing.

### Iodixanol Density Gradient Ultracentrifugation

DGUC was performed as previously described by Karimi et al. (2018) with modifications. OptiPrep^TM^ (60 w/V% iodixanol in distilled water; Axis-Shield, Oslo, Norway) was diluted to 50, 30, and 10% in 0.25 M sucrose buffer (1 mM EDTA and 1mM Tris-HCl, pH 7.4), and a discontinuous gradient was formed by layering 1.33 or 2.66 mL of each solution in 5 mL (referred to as small-scale DGUC) or 10 mL (referred to as large-scale DGUC) polypropylene centrifugation tubes (Beckman Coulter, Pasadena CA, United States). Rat cleared PFP was then layered on to these gradients (0.5 mL on small-scale and 2 mL on large-scale gradients). Afterward, samples were centrifuged in SW55 Ti or Type 70.1 rotors for 24 h at 120,000 ×*g*, 4°C, (Figure 1). Fractions of density gradient layers were collected (F1–F10). From small-scale DGUC the volume of fractions was 500 μL in the case of both F1–F3 and F6–F9, and 330 μL for F4, F5, and F10. From the large-scale DGUC, 1mL fractions were collected uniformly. Samples were used within 24 h or stored at -80°C until use. The density of fractions was calculated based on iodixanol concentration measured by spectrophotometry at 244 nm according to manufacturer’s instruction. Briefly, 10 μL of iodixanol standards with concentrations between 1 and 40% and DGUC fraction samples previously diluted 5,000-fold in distilled water were pipetted into UV microplate wells (Thermo Scientific, Waltham, MA, United States) in triplicates and read by a spectrophotometer at 244 nm (Multiskan GO, Thermo Scientific, Waltham, MA, United States). The density of samples was calculated based on their iodixanol concentration.

### Purification of EV-Rich Fractions by Ultracentrifugation or Size-Exclusion Chromatography

**FIGURE 2:**
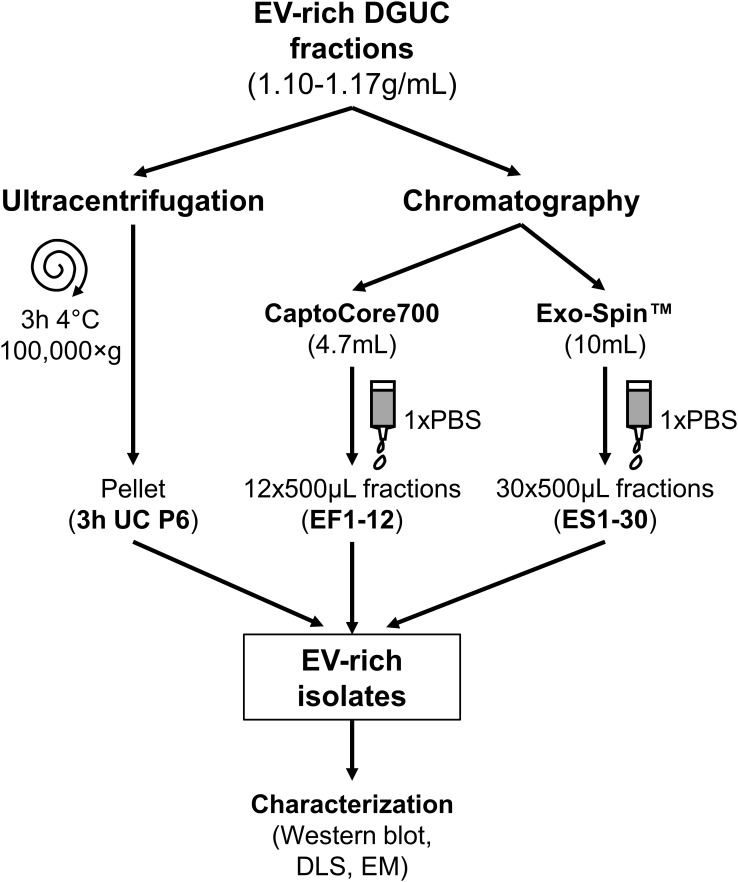
Schematic overview of EV purification methods from DGUC fractions. EV-rich fractions with densities of 1.10–1.17 g/mL underwent bind-elute chromatography by Capto Core 700 column, size exclusion chromatography by Exo-Spin Midi column or classical 3 h pelleting by ultracentrifugation (3 h UC). EV-rich isolates were characterized. EV, extracellular vesicle; DGUC, density gradient ultracentrifugation; P6, pellet from F6; EF, eluted fraction; ES, Exo-Spin fraction; DLS, dynamic light scattering; EM, electron microscopy.

EV-rich fraction of small-scale DGUC, i.e., 300 μL of F6 fraction with highest TSG101 signal was diluted to 5 mL with PBS, then ultracentrifuged for 3 h at 100,000 ×*g*, 4°C (SW55 rotor). Pellets were resuspended in 200 μL PBS (Figure 2).

Bind-elute SEC was performed with modifications to what has been previously described (Corso et al., 2017). EV-rich DGUC fractions (1.5 mL from the pool of F6 + F7 + F8 in case of large-scale DGUC) were loaded onto a HiScreen Capto Core 700 column (4.7 mL bed volume, GE Healthcare Life Sciences). Flow rates and elute conditions were chosen according to the manufacturer’s instructions. Content was eluted with 1× PBS (12 × 500 μL, EF1–12), then the column was washed with 0.1M NaOH in 30% 2-propanol, where CIPs were collected (CIP1-20, 20 × 500 μL; Figure 2). CIP1-20 fractions were immediately pH-adjusted to 7.4 with 0.1M HCl solution in order to assess the composition of bound proteins. Capto Core 700 column was regenerated with 1M NaOH in 30% 2-propanol solution.

Exo-Spin^TM^ gravity elution SEC was performed as previously described (Welton et al., 2015). Pool of DGUC F5-7 (0.7 mL) was loaded onto Exo-Spin^TM^ Midi Columns with 10 mL bed volume (Cell Guidance Systems; Cambridge, United Kingdom). Conditions were chosen according to the manufacturer’s instructions. Thirty EFs were collected (30 × 500 μL, ES1-30; Figure 2).

### Protein Concentration Measurement and Western Blot Analysis

Of each fraction 100 μL sample was mixed with 11 μL of 10× RIPA (Cell Signaling Technology, Danvers, MA, United States) and incubated at 4°C for 5 min. Protein concentration of the samples was determined by bicinchoninic acid assay kit (Thermo Scientific, Waltham, MA, United States). Western blot was conducted as previously described in our laboratory with minor modification (Baranyai et al., 2015). In brief, equal volumes of samples or volumes containing equal amount of protein from each sample were mixed with 1/5 volume of Lane Marker (Thermo Scientific, Waltham, MA, United States) and were loaded on 4–20% Tris-glycine sodium dodecyl sulfate-polyacrylamide gels (Bio-Rad, Hercules, CA, United States), and electrophoresed. Proteins were transferred onto polyvinylidene difluoride membrane (Bio-Rad, Hercules, CA, United States) at 350 mA for 2 h. Membranes were blocked in 5% non-fat milk (Bio-Rad, Hercules, CA, United States) in Tris-buffered saline containing 0.05% Tween-20 (0.05% TBS-T; Sigma, St. Louis, MO, United States) for 2 h at room temperature, and then were probed with primary antibodies overnight at 4°C (primary antibodies: anti-Alix [1:2,000; Cell Signaling, Danvers, MA, United States; 2171S], anti-TSG101 [1:2,000; Abcam, Cambridge, United Kingdom, ab83], anti-CD81 [1:2,000; Santa Cruz Biotechnology, Dallas, TX, United States, sc-7637], anti-Apolipoprotein A1 [1:2,000; Genetex, San-Antonio, TX, United States; GTX112692-100], anti-Apolipoprotein B100/B48 [1:2,000; Merck, Darmstadt, Germany; MABS2046], anti-fibrinogen chain beta (FGB) [1:10,000; Genetex, San-Antonio, TX, United States; GTX54019-100] or anti-albumin [1:10,000; Santa Cruz Biotechnology, Dallas, TX, United States, sc-271605]). After 3 washes in TBS-T, membranes were incubated with corresponding HRP-conjugated secondary antibodies (Cell Signaling, Danvers, MA, United States) for 2 h and washed in TBS-T. Signals were visualized after incubation with enhanced chemiluminescence kit (Bio-Rad, Hercules, CA, United States) by Chemidoc XRS+ (Bio-Rad, Hercules, CA, United States). Image analysis was performed using Image Lab^TM^ software (Bio-Rad, Hercules, CA, United States).

### Dynamic Light Scattering

The size distribution of EVs was measured on a ZetasizerNano (Malvern, United Kingdom) at 20°C using an angle of 173 degrees and 633-nm laser (*n* = 3).

### Transmission Electron Microscopy

Visualization of EVs was accomplished by resin-embedding or the grid-adsorption method by transmission EM.

For resin-embedding EM, samples of interest underwent UC (120,000 ×*g*, 4°C, 1 h) in 5 mL polypropylene centrifugation tubes. The pellets were fixed with 4% paraformaldehyde, postfixed in 1% osmium tetroxide (OsO_4_) for 20 min. After rinsing with distilled water, they were dehydrated by ethanol including block staining with 1% uranyl-acetate in 50% ethanol for 30 min and embedded in Taab 812 (Taab). Ultrathin sections were analyzed with a Hitachi 7100 (Hitachi Ltd, Japan) electron microscope equipped by Veleta, a 4 megapixel side-mounted transmission EM CCD camera (Olympus, Tokyo, Japan). Contrast and brightness of electron micrographs were edited by Adobe Photoshop CS3 (Adobe Incorporation).

For the grid-adsorption method, identical volumes of samples and 1% OsO_4_ in water were mixed and placed onto Formvar coated grids (Agar Scientific; Stansted Mountfitchet, United Kingdom) for 15 min. Following three brief washes in distilled water (3 × 1 min), the excess of the water was removed by touching the edge of the grid to filter paper, and the grids were air-dried and analyzed by transmission EM as described above.

## Results

### Characterization of Fractions Separated by Small-Scale or Large-Scale Density Gradient Ultracentrifugation of Rat Plasma

**FIGURE 3:**
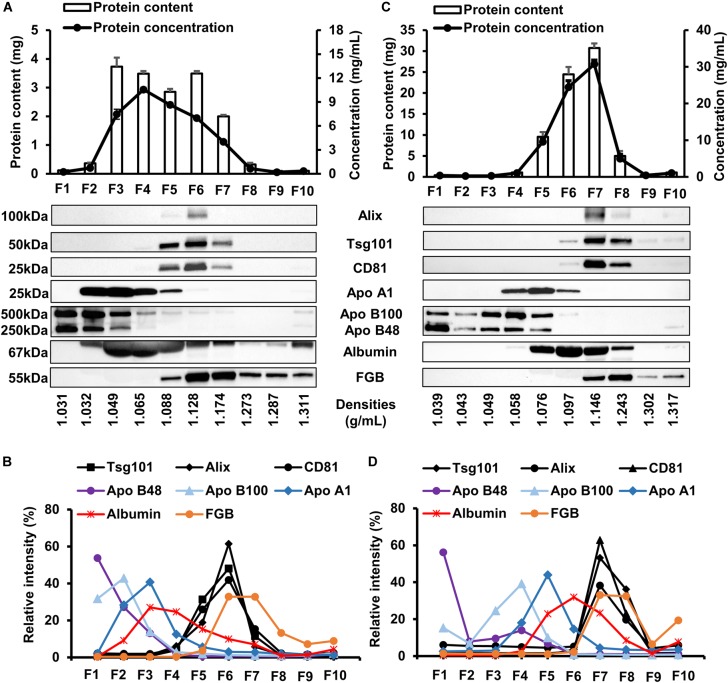
Analysis of protein composition of fractions collected from small-scale and large-scale DGUC. Protein concentration, representative Western blot images and evaluation of each fraction from small-scale **(A,B)** and large-scale **(C,D)** density gradient ultracentrifugation (*n* = 3–6). Data are expressed as mean ± standard error of mean. Relative band intensity was calculated by normalizing individual band intensity to the sum of corresponding total band intensity. F, fraction; FGB, fibrinogen beta chain.

The first set of our experiments were aimed to test whether large-scale or small-scale iodixanol DGUC were suitable for separating EVs from soluble proteins and lipoproteins of the blood plasma. Protein concentration (Figure 3A, line plot) of collected fractions from small-scale DGUC showed a peak in F4 (10.56 ± 0.28 mg/mL; *n* = 3), while the total protein content (Figure 3A, bars) was the highest in F3 (3.73 ± 0.31 mg/mL; *n* = 3) and the lowest in high-density fractions (F8–10; Figure 3A). Protein concentration and total protein content showed different patterns due to the different volumes of the fractions. Equal volumes of F1–10 were loaded for Western blots to analyze the distribution of proteins of interest among fractions from small-scale DGUC, which were then evaluated by normalizing band intensities of each marker to the sum of corresponding band intensities. EV-markers Alix, Tsg101 and CD81 were predominantly observed in F6, at a density of 1.128 g/mL (61.5 ± 10.35%; 48.1 ± 5.8%; 41.9 ± 3.8%, respectively), while a smaller amount of markers was detected in F5 (18.9 ± 4.1%; 31.3 ± 4.3%; 25.9 ± 2.9%) and F7 (5.8 ± 1.6%; 11.5 ± 2.2%; 15.4 ± 1.1%; Figures 3A,B). In addition, EV-markers could be detected in F5 to F10 if samples with equal amount of protein (10 μg) were loaded for Western blots, indicating the presence of EV populations at higher densities (1.174–1.287 g/mL; Supplementary Figure 1). After identifying EV markers, we also tested markers for contaminants such as lipoproteins and abundant plasma proteins. Chylomicron-related Apo B48, LDL-related Apo B100 and HDL-related ApoA1 were mainly present in low-density fractions F1–4 (95.5 ± 3.5, 91.4 ± 2.8% and 83.8 ± 1.8%; *n* = 3), but a low proportion of total Apo A1 was detected in EV-rich fractions as well (Apo A1: 11.4 ± 1.2%; Apo B100: 1.35 ± 0.45; Apo B48: 0.63 ± 0.03; in F6; Figures 3A,B). A moderate amount of albumin signal was observed in F6 (9.91 ± 0.4%; *n* = 3), the majority of albumin signals were detected in the lower density fractions F1–5 (76.44 + 1.1% of total signal). Despite the efficient separation of lipoprotein and albumin from EVs, fibrinogen (FGB) content of F6 was significant (32.84 ± 1.53% of total signal; *n* = 3; Figures 3A,B).

**FIGURE 4:**
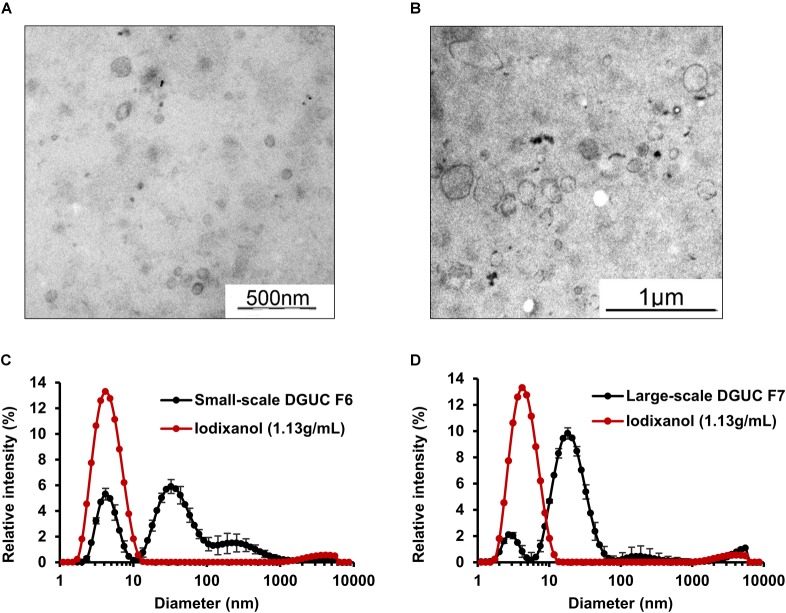
Morphological analysis of extracellular vesicles isolated by small-scale or large-scale DGUC. Representative resin-embedding transmission electron microscopy images on extracellular vesicles from small-scale DGUC F6 **(A)** and large-scale DGUC F7 **(B)**. Dynamic light scattering measurement was performed from the small-scale **(C)** and large-scale **(D)** EV-rich fractions in parallel with corresponding control iodixanol solution (*n* = 3–6) Data are expressed as mean ± standard error of mean. DGUC, density gradient ultracentrifugation; F, fraction; EV, extracellular vesicle.

Samples from small-scale DGUC F6 and F7 were analyzed using EM to visualize isolated EVs. To exclude the interference of iodixanol with EM analysis, a control solution (20 w/V% iodixanol in distilled water) was also examined (Supplementary Figure 2A). Membrane-enclosed vesicles with a diameter of 48–310 nm could be visualized with EM in F6 and F7 (*n* = 25; Figure 4A). Selected fractions were examined by DLS to assess the size distribution of vesicles, as well. DLS analysis of fractions established that F6 contained mainly small-sized vesicles (mean diameter: 38 ± 2 nm; *n* = 6), but a significant peak was also observed at 4nm, likely due to the iodixanol content of the samples (Figure 4C). Peaks with a diameter of 4 and 5,000 nm corresponding to iodixanol were also present in DGUC fractions (Figures 4C,D and Supplementary Figure 2).

DGUC was also tested at a larger scale to increase the loadable sample volume and EV yield. 2 mL of plasma was layered onto the top of total 8 mL of iodixanol gradient (Figure 1). Highest protein content was observed in F6 and F7 in large-scale DGUC (24.17 ± 0.99 mg, 30.67 ± 1.54 mg; *n* = 6; Figure 3C). EV-markers Alix, Tsg101 and CD81 were detected in F7 at 1.146 g/mL (38.2 ± 13.9%; 53.2 ± 0.6%; 62.8 ± 23.1%; *n* = 2–4), while a small proportion of markers were observed in F6 and F8 (5.2 ± 1.4%, 3.6 ± 1.1% and 2.5 ± 0.6% in F6; 19.8 ± 7.7%; 36.2 ± 2.0%; 23.1 ± 5.9% in F8; *n* = 2–4). Still, a significant amount of albumin was found in both F6 and F7 (55.2 ± 1.6%; *n* = 3). Additionally, FGB was also present in F7–8 (65.5 ± 3.0%; *n* = 3). The majority of Apo B48 and B100 were separated from EV-rich F6–8 (3.3 ± 0.06% and 2.6 ± 0.1%; *n* = 3), but a small proportion of ApoA1 was observed in fractions F6 (3.01 ± 1.29%; *n* = 3; Figures 3C,D). Between DGUC fractions of fresh- or frozen plasma there was no substantial difference in the distribution of EV markers or contaminants (see Supplementary Figure 6). By using EM and DLS analysis, the presence of vesicles in F7 was confirmed; EVs in various size were identified by EM (Figure 4B), while DLS evidenced the presence of smaller particles in F7 (mean diameter: 18 ± 10 nm, *n* = 3). Peaks with a diameter of 4 and 5,000 nm corresponding to iodixanol were also present in F7 of large-scale DGUC (Figure 4D).

### Purification of EV-Rich Fractions of Small-Scale DGUC by 3 h Ultracentrifugation

The fraction F6 of the small-scale DGUC, which showed the highest EV-content according to our Western blot analysis, was diluted approximately 17-fold and centrifuged for 3 h at 100,000 ×*g*, to reduce the remaining plasma contaminants and iodixanol in the EV-rich fraction, then the pellet was analyzed (Figure 2). UC for 3 h yielded notably less protein in pellets (P6) compared to original DGUC fraction (109.4 ± 22.2 μg in P6 vs. 3,475.9 ± 249.8 μg in loaded F6; *n* = 6; Supplementary Figure 3A), while the iodixanol concentration was under the detection limit in resuspended pellets as measured by spectrometry at 244 nm. A high number of EVs was visualized in P6 with EM (Supplementary Figure 3B), however, comparison between F6 and P6 by Western blot showed that the band intensity of EV markers was considerably lower after 3 h UC as compared to that of corresponding F6 (Alix: 29.3 ± 0.2%; Tsg101: 24.4 ± 2.4%; CD81: 16.5 ± 1.2%; *n* = 3). In addition, signals of albumin and lipoprotein markers were also reduced, but a high amount of FGB was still present in the pellet as compared to EVs or other contaminants (albumin: 10.1 ± 1.7%; ApoA1: 24.3 ± 5.9%; Apo B48: 24.4.0 ± 2.2%; Apo B100: 17.0 ± 3.8%; FGB: 53.1 ± 2.1%; *n* = 3; Supplementary Figures 3C,D). Furthermore, a significant amount of vesicles was also detectable in the supernatant by Western blot obtained after 3h UC evidencing the low efficiency of pelleting EVs by 3 h of UC (Supplementary Figure 3E).

### Purification of EV-Rich Fractions of Large-Scale DGUC by HiScreen CaptoCore700 Bind-Elute Size-Exclusion Chromatography

**FIGURE 5:**
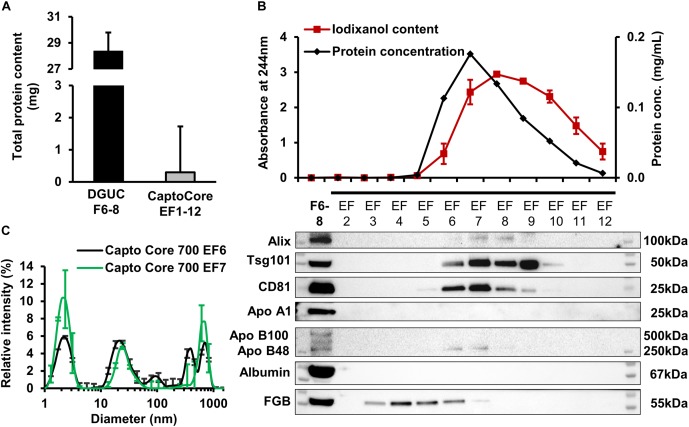
- Analysis of Capto Core 700 elution fractions. Average of total protein content **(A)**, protein and iodixanol concentration and representative Western blot images **(B)** from EF2–12 with corresponding loaded DGUC fractions F6–8 (*n* = 3). Dynamic light scattering measurement **(C)** was performed from EF6–7 (*n* = 3). Data are expressed as mean ± standard error of mean. DGUC, density gradient ultracentrifugation; EF, eluted fraction.

The applicability of BEC using HiScreen Capto Core 700 columns for further purification of EV-rich DGUC fractions was also studied. Since the column has high binding capacity for proteins (13 mg ovalbumin/mL of resin, Corso et al., 2017), and since our preliminary experiments suggested a high loss of EVs if samples with lower volume and protein concentration are applied onto this column (data not shown), 1.5 mL pool of EV-rich fractions F6, F7, and F8 of large-scale DGUC with total protein contents of 28.36 ± 1.43 mg were loaded onto the column. Then 12 eluted fractions (EF1-12) were analyzed. The total protein amount in EF fractions was lower as compared to the loaded large-scale DGUC fractions (295.3 ± 121.3 μg eluted vs. 28.4 ± 1.4 mg input) (Figure 5A). The majority of signals for Alix, Tsg101 and CD81 were found in EF6-9. Albumin and Apo B100 were under detection limit in EF1–12, however, FGB was detectable in EV-containing fractions EF2–EF6 (Figure 5B). DLS analysis of fractions established that EF6 and EF7 contained small-sized vesicles (mean diameter: 24.4 ± 5.1 nm, *n* = 3; and 24.4 ± 4.9 nm, *n* = 3), and the iodixanol-specific peaks (mean diameter: 4 and 5,000 nm) were also present (Figure 5C and Supplementary Figure 2). In order to remove bound proteins, the column was washed using 0.1M NaOH in 30% 2-propanol, and CIP fractions were collected (CIP1-20). CIP5-15 contained the highest protein concentrations, and therefore, were chosen for Western blot to identify the composition of proteins bound by the column. CIP fractions included high amounts of albumin and FGB, but EV markers were also detectable, thus indicating that the column retained significant amount of EVs as well (Supplementary Figure 4).

### Purification of EV-Rich Fractions by Exo-Spin^TM^ Size-Exclusion Chromatography

Classical SEC by Exo-Spin^TM^ was also evaluated for EV-isolation. A pool of small-scale DGUC fractions F5–7 with the highest EV-content (700 μL with 4.8 ± 0.3 mg total protein) was loaded onto Exo-Spin^TM^ columns with 10 mL bed volume. Then 30 fractions of 500 μL were collected (ES1-30). Proteins were found in fractions from ES10, while maximum protein concentration was observed in ES18 (0.91 ± 0.02 mg/mL). In addition, significant amount of iodixanol was only detected in ES17 (Supplementary Figure 5A). According to Western blot analysis of ES8–18, EV-markers were present in ES12–18 with significant amounts of impurities (Supplementary Figure 5B).

## Discussion

Here we described an isolation method for EVs from fresh or frozen blood plasma based on iodixanol DGUC followed by BEC. The current method yields EVs in higher amounts than previously described isolation techniques, such as UC and SEC, without lipoprotein and albumin contamination. However, we also report that fibrinogen may be a significant contaminant in EV isolates from blood plasma. This is the first demonstration that isolation of lipoprotein- and albumin-free EVs from blood plasma can be achieved by DGUC followed by BEC. This comes at the expense of reduced EV yield, however.

Since the efficiency of discontinuous iodixanol DGUC used alone, or in combination with prior UC or filtration was low or has not been determined (Kalra et al., 2013; Karimi et al., 2018), here we optimized iodixanol DGUC by reducing the ratio of gradient to sample volume and by increasing UC time. As a result, our small-scale DGUC protocol separated the majority of EVs from the vast majority of lipoproteins with a high efficiency (42–62% of EVs co-isolated with only 1–3% of lipoproteins in F6), which would lead to a significant improvement in the reliability of downstream analyses (Sluijter et al., 2018). We also report that our DGUC approach reduced the amount of soluble protein contaminants, i.e., albumin and fibrinogen, which have been identified as some of the most abundant contaminants of EV preparations (Baranyai et al., 2015; Foers et al., 2018; Karimi et al., 2018). Nevertheless, we identified a small proportion of EVs at higher densities, confirming previous findings (Bobrie et al., 2012), suggesting that composition, hence function of a minority of EVs in the blood plasma might be substantially different from that of traditionally studied EV populations.

Additionally we investigated the effect of increased gradient to sample ratio on separation efficiency. Large-scale DGUC showed a separation pattern similar to our small-scale DGUC protocol. However, the resolution was reduced as higher proportion of albumin and HDL contamination was observed in the EV-rich fractions, which is in contrast to findings of a previous study (Karimi et al., 2018). This finding may result from the different type of rotors in small- and large-scale DGUC experiments. The difference in gradient formation between the applied swing-out and fixed angle rotor may affect the migration, distribution and composition of EV subpopulations in the gradient. Also, the differences in separation pattern may arise from the diluting effect of the increased volume of blood plasma (density: 1.025 g/mL) on the high-density fractions which may reduce separation efficiency and shift fractions (Steensgaard et al., 1975). Thus we can conclude that DGUC is scalable only by maintaining gradient to sample ratios of approximately 8:1.

Furthermore, our results also confirm that not only lipoproteins, but aggregates of soluble plasma proteins may appear at a wide range of buoyant densities, as shown before (Aatonen et al., 2014). Our data suggests that separation methods relying solely on differences in density may not be suitable for EV isolation from blood and that additional purification steps may be necessary since impurities with high binding capacities for various biological substances and chemicals, such as albumin, may significantly alter the results of functional- and *in vivo* studies on EVs. We can thus see that the 24 h-long DGUC is applicable as a first-step method for the isolation of EVs from blood plasma to reduce lipoprotein- and proteinaceous contaminations.

Three different methods were tested for second-step purification of EV-rich fractions from DGUC in order to increase the purity of our isolates. Firstly, classical purification by pelleting UC for 3 h was performed on small-scale DGUC fractions to avoid co-isolation with lipoproteins. Recovery of EVs after 3 h UC was low, which is in accordance with our previous observations (Baranyai et al., 2015) and reports by others (Momen-Heravi et al., 2012; Vergauwen et al., 2017). UC decreased the amount of albumin, however, the relative fibrinogen content increased, suggesting that fibrinogen may tend to form high-density aggregates or fibrin fibers during the isolation procedure which would further limit the applicability of UC as a second-step purification method.

We also tested a commercially available SEC column for the further purification of EV-rich DGUC fractions. We observed that no further purification from lipoproteins or proteins was achieved by this method, which is in accordance to previous findings by our workgroup and others, showing that only a minor fraction of EVs isolated from rat blood plasma could be separated from albumin by SEC (Boing et al., 2014; Baranyai et al., 2015; de Menezes-Neto et al., 2015). In addition, heterogeneity of protein contaminants of EV isolates obtained from blood by DGUC followed by SEC has been assessed inadvertently by a proteomic analysis by Karimi et al. (2018) who detected several hundred non-vesicular protein entities in their EV preparations, the abundance of which in several cases even exceeded that of the EV-associated proteins. These data suggest that SEC products currently available may not be applicable in workflows to increase purity of EV-rich fractions from DGUC. However, since our results indicate that EVs from rat plasma have a lower average diameter as compared to human EVs (Baranyai et al., 2015), certain SEC matrices may be useful for the isolation of EVs from the blood of different species.

Bind-elute chromatography with Capto Core 700 columns was applied to our study successfully for the first time in the literature to further purify EV-rich DGUC fractions, confirming previous results of Corso et al. (2017) on EVs isolated from cell culture supernatant and displaying the feasibility of the use of BEC as a second-step for EV purification from blood plasma. BEC yielded approximately three-fold higher amount of proteins than 3 h UC without significant dilution where contaminating albumin and ApoA1 signals were under the detection limit, resulting in EVs in sufficient amount for rodent *in vivo* experiments. However, since input EV signals were significantly higher than that of in the separated EV fractions, our findings suggest that Capto Core 700 retained not only small molecules, but also a fraction of EVs as well. This shows that the efficiency of this method may require further refinement. Furthermore, the successful separation of EVs from albumin and ApoA1 suggests that they may not be integral parts of EV proteome, but contaminants in EV preparations which are difficult to remove. In contrast, and in accordance with previous findings with different isolation methods (Aatonen et al., 2014; Roura et al., 2018), fibrinogen was detected in early EV-rich fractions after purification on Capto Core 700. This suggests that fibrinogen may have a strong interaction with certain EV subpopulations, or it could also be hypothesized that fibrinogen contamination is partially caused by coagulation during isolation, since EVs and particles have been shown to possess pro-coagulant properties (Siljander et al., 1996). To lower or remove fibrinogen contamination, further investigation is needed.

We found that in fractions of large- and small-scale DGUC and BEC EV subpopulations with distinct marker patterns are enriched. This phenomenon was described in other papers previously. For example, in one of our earlier studies we observed that a certain population of EVs that were positive for TSG101 but not for CD63 eluted later than double positive vesicles when separated by SEC on Sephacryl S400 from rat blood plasma (Baranyai et al., 2015). Elsewhere, Western blot analysis using several EV markers demonstrated the separation of subpopulations of EVs from blood by iodixanol gradient UC and SEC (Karimi et al., 2018). Similar results were obtained by UC of cell supernatants (Jeppesen et al., 2014), suggesting variable sedimentation characteristics and marker composition of EV subpopulations.

Our current results indicate that while certain high-abundance contaminants could be removed from EV isolates, some components of the blood plasma cannot be separated from EVs by currently available technologies with reasonable efforts. Therefore, it is plausible that for different downstream analyses, different EV isolation methods must be applied, since certain studies may require higher purity EVs, but others may tolerate the presence of certain contaminants. Nevertheless, despite the remaining contaminants, e.g., fibrinogen, EV isolates obtained by the current method have the potential applicability *in vitro* and *in vivo* non-clinical studies with appropriate control groups to distinguish the effects of EVs and contaminants. EVs isolated by our method may advance studies on use of EVs in tumor therapy (Rolfo et al., 2014; Lam et al., 2015) or tissue repair, e.g., after cardiac ischemia/reperfusion injury (Giricz et al., 2014; Barile et al., 2017; Sluijter et al., 2018).

This study faces certain limitations. We have not evaluated the exact efficiency of EV isolation by Capto Core 700 due to the high difference between loaded and yielded protein content. Here we assessed only albumin and fibrinogen as protein contaminants of EV isolates. Nevertheless, since a multitude of other contaminants have been identified in EVs obtained by multi-step methods (Karimi et al., 2018), determining the presence or concentration of certain other contaminants may also be necessary for specific downstream applications of the EV isolates. Furthermore, investigation on functionality of EVs obtained by the current method has not been performed.

## Conclusion

In summary, we report an EV isolation method from blood plasma based on iodixanol DGUC which is able to minimize lipoprotein contamination while keeping high separation efficiency. Although further purification of EV-rich DGUC fractions by BEC to remove soluble blood components results in EVs devoid of albumin and lipoproteins, but at the expense of yield. Furthermore, certain blood plasma components still remain in EV isolates obtained by DGUC and BEC. Therefore, it is likely that for specific application of EV-rich fractions, development of individual EV isolation methods should be considered.

## Availability of Data and Materials

The datasets used and/or analyzed are available from the corresponding author on reasonable request.

## Author Contributions

ZO participated in study designing and performed experiments, evaluated results, and drafted the manuscript. CP, GB, and LA performed the experiments and interpreted the data. CN and MM-K performed DLS measurement. ÁK performed EM analysis and provided professional advice. PF and EB revised the manuscript, the intellectual content and provided professional advice. ZG designed experiments, wrote manuscript, revised the intellectual content, and provided professional advice. All authors read and approved the final manuscript.

## Conflict of Interest Statement

PF is the founder and CEO and ZG is involved in the management of Pharmahungary Group, a group of R&D companies, the pipeline of which includes development of EV isolation techniques (www.vezics.com). The handling Editor is currently organizing a Research Topic with one of the authors PF, and confirms the absence of any other collaboration. The remaining authors declare that the research was conducted in the absence of any commercial or financial relationships that could be construed as a potential conflict of interest.

## Abbreviations

Apo B100/48: apolipoprotein B100/48
ApoA1: apolipoprotein A1
BEC: bind-elute chromatography
CIP: clean-in-place fraction
DGUC: density gradient ultracentrifugation
DLS: dynamic light scattering
EF: eluted fraction
EM: electron microscopy
ES: Exo-Spin fraction
EV: extracellular vesicle
F: fraction
FGB: fibrinogen beta chain
P6: pellet
PFP: platelet-free plasma
RIPA: radio immunoprecipitation assay buffer
S6: supernatant
SEC: size-exclusion chromatography
UC: ultracentrifugation
