# Supplementary material for: Isolation of High-Purity Extracellular Vesicles by the Combination of Iodixanol Density Gradient Ultracentrifugation and Bind-Elute Chromatography From Blood Plasma
Source: Front Physiol. 2018 Oct 23;9:1479. doi: 10.3389/fphys.2018.01479 (PMC6206048; doi:10.3389/fphys.2018.01479)
Supplement: Supplementary file 1 [file Data_Sheet_1.pdf]

Supplementary Material

Isolation of high-purity extracellular vesicles by the combination of iodixanol density gradient ultracentrifugation and bind-elute chromatography from blood plasma

Zsófia Onódi[1], Csilla Pelyhe[1], Csilla Terézia Nagy[1], Gábor B. Brenner[1], Laura Almási[1], Ágnes Kittel[2], Mateja Manček-Keber[3,4], Péter Ferdinandy[1,5], Edit I. Buzás[6], Zoltán Giricz[1,5,*]

*** Correspondence:**
Corresponding author: Zoltán Giricz PharmD PhD
address: H-1089 Budapest, Nagyvárad tér 4. Hungary
e-mail: giricz.zoltan@med.semmelweis-univ.hu
Tel: +3612104416
Fax: +3612104412

Supplementary Figure 1

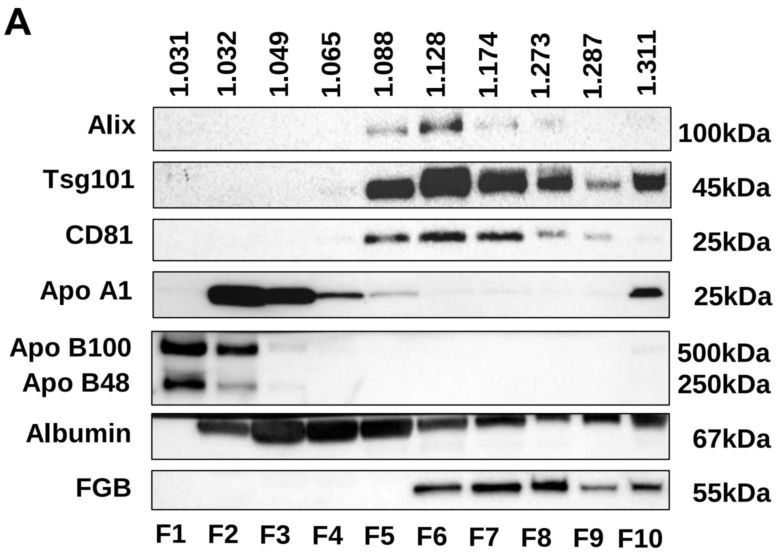

Supplementary Figure 1 - Representative images from equal protein (10µg) loading Western blot from small-scale DGUC fractions. DGUC: density gradient ultracentrifugation; FGB: fibrinogen beta chain

Supplementary Figure 2

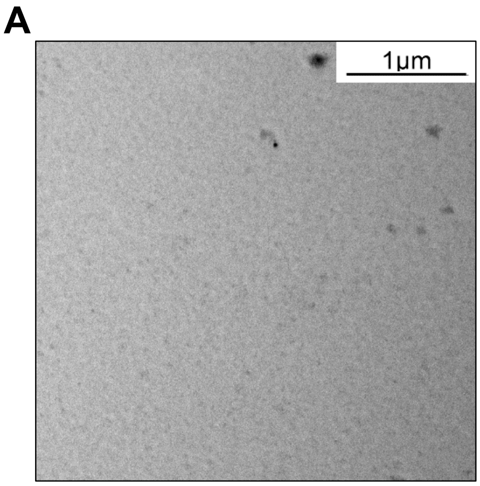

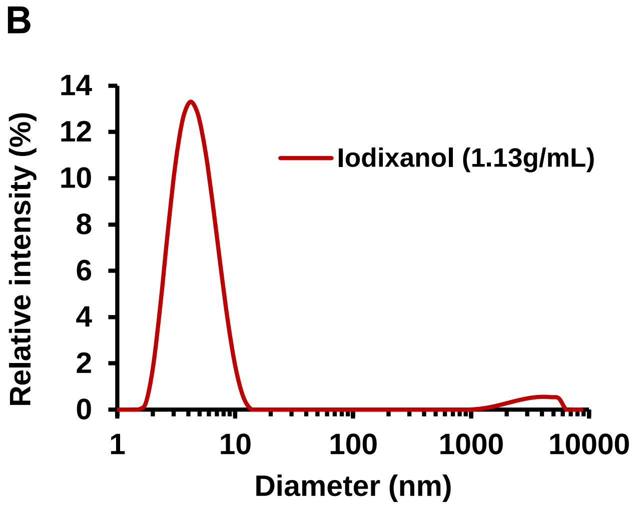

Supplementary Figure 2 - Analysis of 1.13mg/mL iodixanol solution. Representative electron microscopy image, sample prepared by grid-adsorption method (A) and dynamic light scattering measurement (B) from control iodixanol solution (~20 w/V%, 1.13g/mL)

Supplementary Figure 3

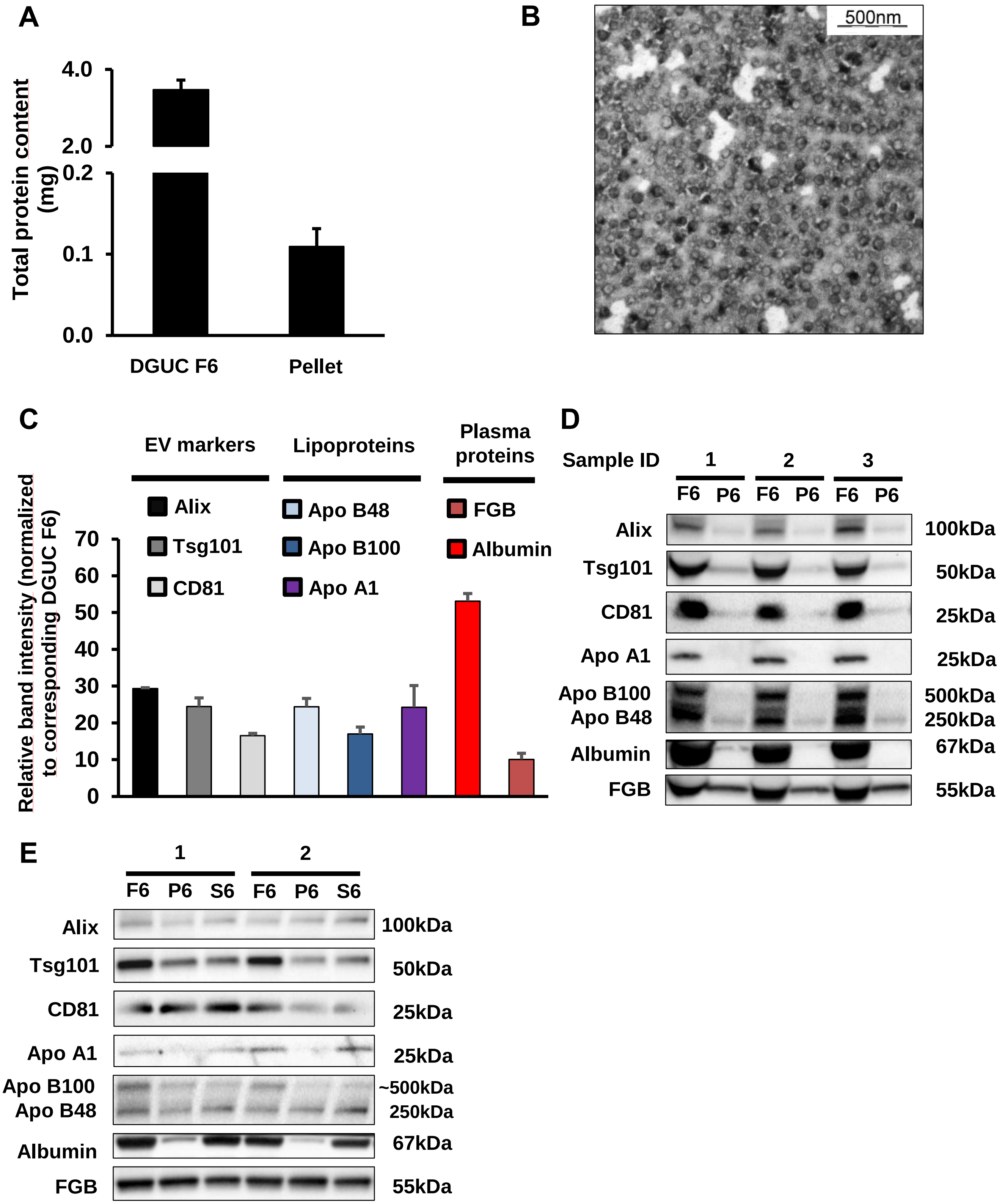

Supplementary figure 3 – Analysis of 3h ultracentrifugation of small-scale DGUC F6. Average of protein content (A), representative electron microscopy image, sample prepared by grid-adsorption method (B) and evaluation of Western blots (C) of 3h UC pellet (P6) compared to corresponding DGUC F6. Evaluation (C) and representative images (D) from Western blot comparison of F6 to P6. Equal protein loading Western blot of F6,

P6 and S6 (D; n=3). Data are expressed as mean ± standard error of mean. DGUC: density gradient ultracentrifugation; F: fraction; P6: pellet from F6; S6: supernatant from F6; FGB: fibrinogen beta chain

__Supplementary Figure 4__

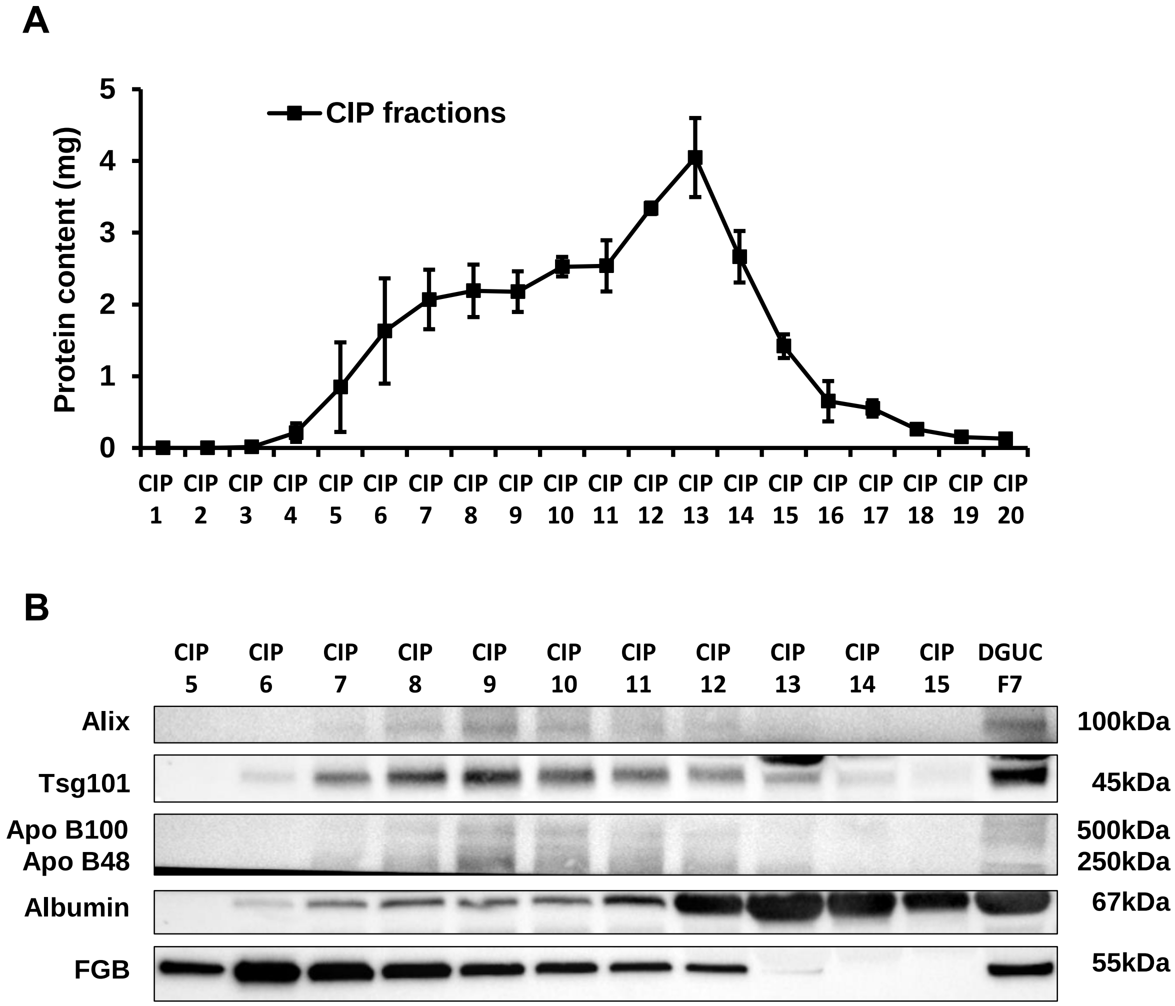

Supplementary figure 4 – Analysis of eluted CIP fractions from Capto Core 700 column. Average of protein content (A) of eluted CIP fractions. Representative Western blot images from CIP5-15 and DGUC F7 (B; n=2). Data are expressed as *mean ± standard error of mean*. CIP: cleaning-in-place; DGUC: density gradient ultracentrifugation; F: fraction; FGB: fibrinogen beta chain

Supplementary Figure 5

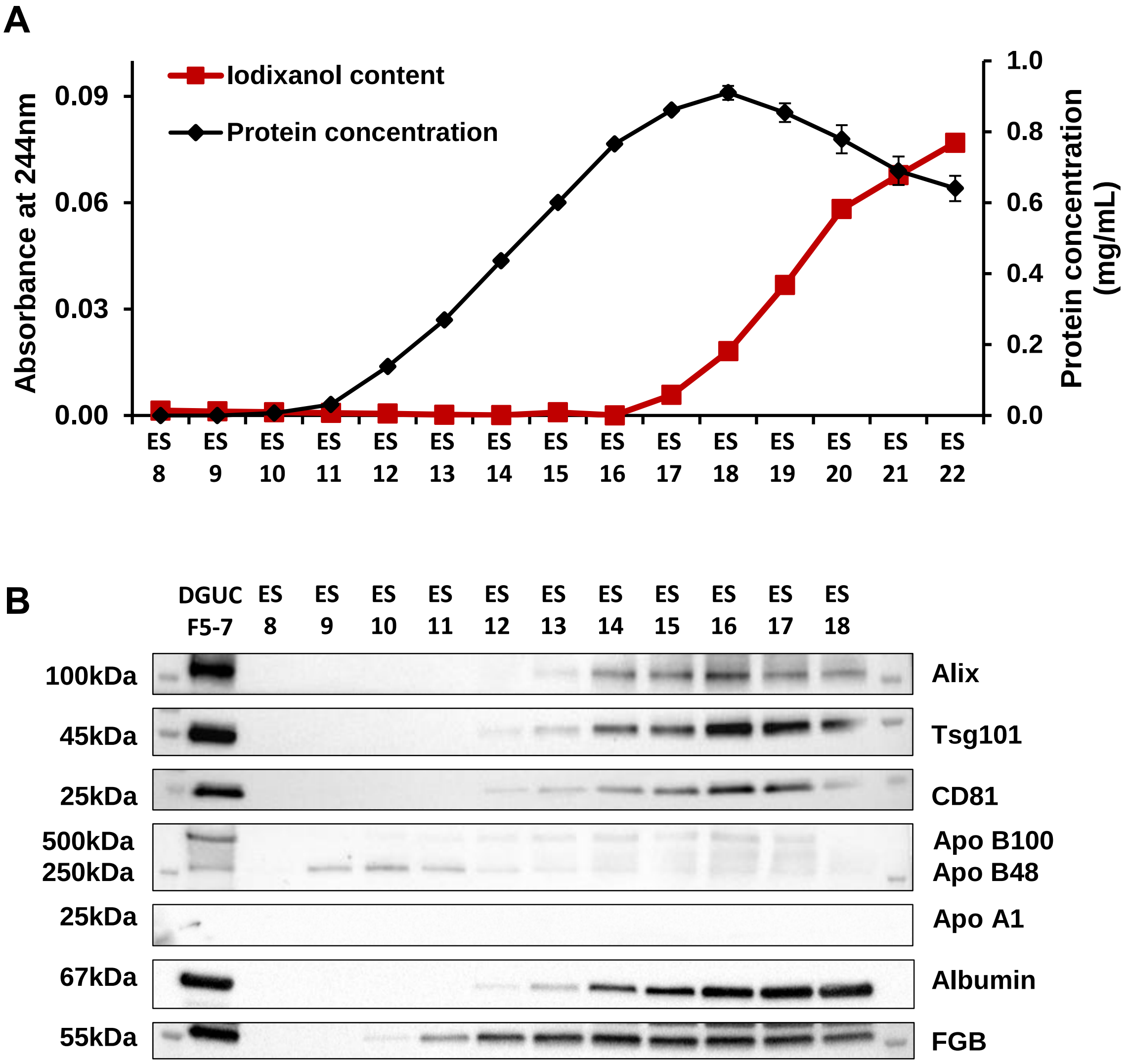

Supplementary figure 5 – Analysis of Exo-Spin™ column-based EV-isolation from small-scale DGUC F5-7. Average of protein content (A) and representative Western blot images (B) of Exo-Spin™ isolation compared to corresponding DGUC F5-7. Data are expressed as mean ± standard error of mean. ES: Exo-Spin fration; DGUC: density gradient ultracentrifugation; F: fraction; FGB: fibrinogen beta chain

Supplementary Figure 6

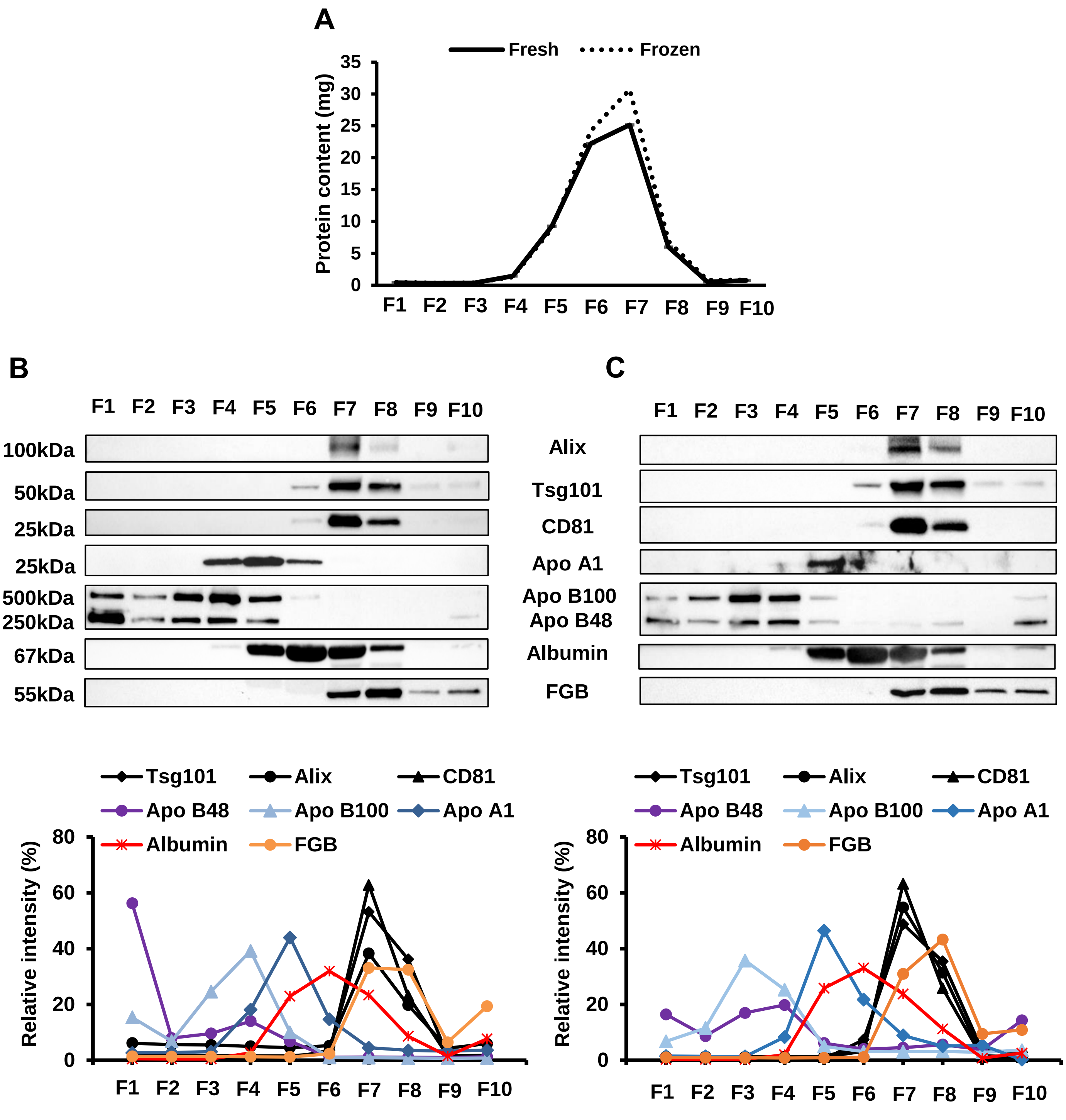

Supplementary figure 6 – Comparison of EV isolation from fresh and frozen plasma by large-scale DGUC (Type 70.1 rotor) Average of protein content of fresh and frozen (data from Fig.3. C) samples (A) and representative Western blot images of frozen (data from Fig.3 C and D) (B), and fresh (C) EV isolates by large-scale DGUC. Data are expressed as mean ± standard error of mean. DGUC: density gradient ultracentrifugation; F: fraction; FGB: fibrinogen beta chain
