# Supplementary material for: Isolation of High-Purity Extracellular Vesicles by the Combination of Iodixanol Density Gradient Ultracentrifugation and Bind-Elute Chromatography From Blood Plasma
Source: Front Physiol. 2018 Oct 23;9:1479. doi: 10.3389/fphys.2018.01479 (PMC6206048; doi:10.3389/fphys.2018.01479)
Supplement: Supplementary file 2 [file Data_Sheet_2.pdf]

Supplementary Methods

Isolation of high-purity extracellular vesicles by the combination of iodixanol density gradient ultracentrifugation and bind-elute chromatography from blood plasma

Zsófia Onódi[1], Csilla Pelyhe[1], Csilla Terézia Nagy[1], Gábor B. Brenner[1], Laura Almási[1], Ágnes Kittel[2], Mateja Manček-Keber[3,4], Péter Ferdinandy[1,5], Edit I. Buzás[6], Zoltán Giricz[1,5, *]

*** Correspondence:**

Corresponding author: Zoltán Giricz PharmD PhD

address: H-1089 Budapest, Nagyvárad tér 4. Hungary

e-mail: giricz.zoltan@med.semmelweis-univ.hu

Tel: +3612104416

Fax: +3612104412

Supplementary materials and methods

Equipment, consumables

1. **Sample collection**
   - BD Vacutainer ACD tubes
2. **For sample preparation and iodixanol density gradient ultracentrifugation:**
   - Centrifuges for sample tubes and blood collection tubes
   - Ultracentrifuge (e.g., Beckman L7-55)
   - Ultracentrifuge rotors, SW 55 or Type 70.1 (Beckman)
   - 5mL or 10mL polypropylene tubes for ultracentrifugation (UC tubes, Beckman)
   - 1mL pipet and tips
   - 0.1µm filters (Durapore PVDF, Millex)
   - 1.5mL sample tubes
   - 15mL and 50mL sample tubes
3. **For Capto Core 700 bind-elute chromatography**
   - HiScreen Capto Core 700 column, 4.7mL bed volume (GE Health)
   - Peristaltic or syringe pump
   - Vacuum pump for degassing solutions
   - Silicone tubing
   - 1mL and 50mL syringes
   - 0.1µm filters (Durapore PVDF, Millex)
   - 1.5mL sample tubes
4. **For ExoSpin™ size-exclusion chromatography**
   - ExoSpin™ size-exclusion chromatography column, 10mL bed volume (Cell Guidence System)
   - 1mL pipette and tips
   - 1.5mL sample tubes

Reagents

1. **For iodixanol density gradient ultracentrifugation:**
   - 60% iodixanol in distilled water (OptiPrep™, Axis-Shield)
   - 50, 30 and 10 w/V% iodixanol solutions
   - Working solution: 0.25M sucrose, 6mM EDTA, 60mM Tris-HCl; pH 7.4
   - Homogenization medium: 0.25M sucrose, 1mM EDTA, 10mM Tris-HCl; pH 7.4
2. **For Capto Core 700 bind-elute chromatography**
   - phosphate buffer saline (PBS); pH 7.4
   - 0.1M NaOH in 30% 2-propanol (optional)
   - 1M NaOH in 30% 2-propanol
   - 20% ethanol in PBS
3. **For ExoSpin™ size-exclusion chromatography**
   - PBS; pH 7.4

Prepare iodixanol solutions according to manufacturer’s instructions.

Filter all buffers on 0.1µm filters before use.

Protocol

1. Sample collection and preparation

1.1. Collect venous blood from anesthetized animals into Citrate Dextrose A vacuum tubes. Do not shake. Put blood on ice. Store prepared plasma samples on ice until use. Use within 30 min.
1.2. Centrifuge fresh whole blood at 2,500 rcf for 15min at 4 °C.
1.3. Transfer the supernatant in another 15 mL tube, and centrifuge again at 2,500 rcf for 15min at 4 °C.
1.4. Transfer carefully the supernatant in another 15 mL sample tube. Centrifuge the supernatant at 18,000 rcf for 90min at 4°C.
1.5. Collect supernatant into a new 15mL Falcon tube. This fraction will be used for EV isolation.

2. Iodixanol density gradient ultracentrifugation in SW55 or T70.1 rotors

2.1. Prepare your Working solution and Homogenization medium. Set the pH of both solutions to 7.4 and filter them with 0.1μm filter before use.
2.2. Cool UC buckets or rotor to 4°C.
2.3. If plasma samples were frozen, centrifuge thawed samples on 2,500 rcf at 4°C for 5min before use.
2.4. Prepare iodixanol solutions. Cover each iodixanol-containing tubes and store at 4°C until use.
2.5. Carefully layer iodixanol solutions to each other according to the following steps:
2.5.1. Mark your tubes to allow identification of samples.
2.5.2. Layer 1.33mL (small-scale; SW55) or 2.66mL (large-scale; T70.1) 50% iodixanol solution on the bottom of UC tube.
2.5.3. Then, take UC tube and 1mL pipette, and hold the tube angled (approx. 20-40°) by hand (Fig.1).

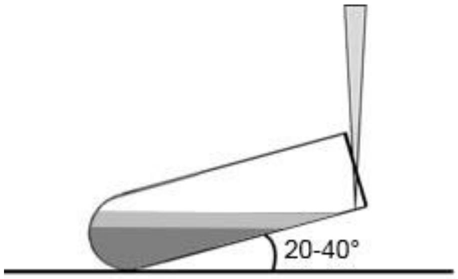

Figure 1 – Correct position of tube during layering gradients

2.5.4. Start pipetting 1.33mL (small-scale; SW55) or 2.66mL (large-scale; T70.1) of 30% iodixanol solution on the top of the 50% solution very slowly and carefully. Do not touch the top of the previous layer.
2.5.5. After the 30% layer, start pipetting carefully 1.33mL (small-scale; SW55) or 2.66mL (large-scale; T70.1) of 10% iodixanol solution on the top of the 30% solution without repositioning the tube.
2.5.6. Slowly turn the tube back to vertical position. NOTE: If layering was careful enough, you must see thin borders ("swimming discs") between each layer (Fig.2A). If you do not see these "discs", layers started diffusing to each other and your gradients is not

discontinuous anymore (Fig.2B). In case of that, discard gradient and use another tube and layer new gradients.

2.5.7. Angle the tube to approximately 60° and layer 0.5mL plasma onto the gradients carefully. NOTE: Be very careful during handling filled tubes, avoid sudden moves to prevent diffusion of gradients.

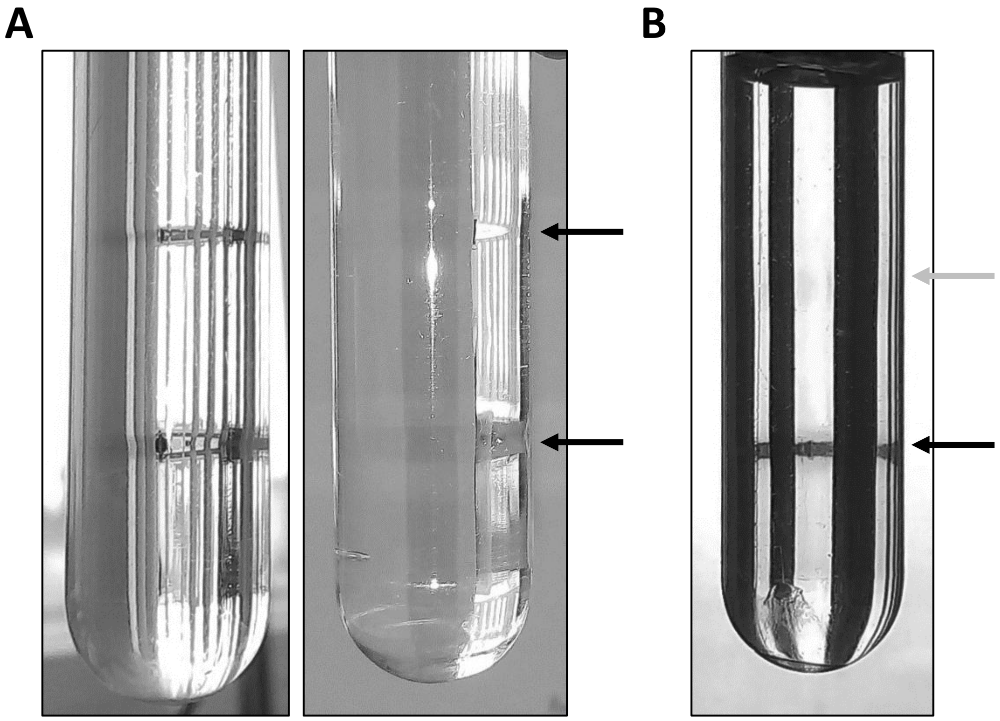

Figure 2 – Correct (A) and damaged (B) iodixanol gradients

Black arrow: good layering, sharp borders; gray arrow: diffused border

2.6. Put the filled UC tubes into precooled buckets or rotor.

2.7. Balance the whole buckets (SW55) or tubes (T70.1) including their lids and filled tubes within 10mg differences between opposite buckets or tubes. If the difference between the two buckets or tubes is larger, use the remnaining plasma to balance.

2.8. Ultracentrifuge samples at 4°C for 24h. For SW55 rotor: use 35,500rpm, for Type 70.1 rotor use 41,000rpm.

2.9. Carefully remove buckets or tubes from rotor. Open buckets, and check the appearance of layers by eye.

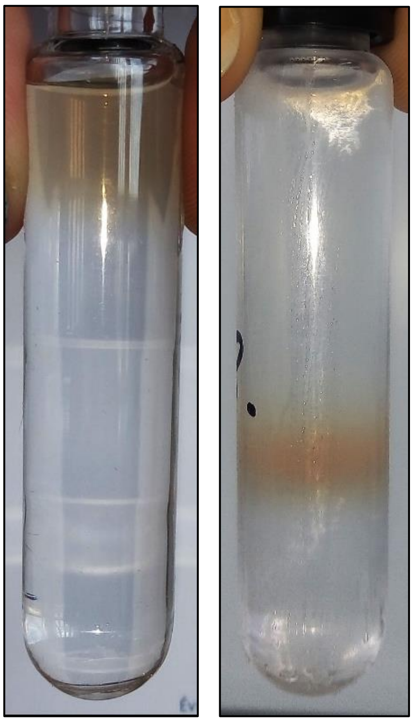

Figure 3 – Density gradient tubes before (left) and after (right) 24h ultracentrifugation

2.10. Collect 10 fractions from top to the bottom by 1mL pipette. Pipet very slowly by only touching the very top of liquid by the pipet tip.
2.11. Fraction volumes in case of SW55: F1-3 – 500µL, F4-5 – 330µL, F6-9 – 500µL, F10 – 330µL
2.12. Fraction volumes in case of T70.1: F1-10 – 1mL
2.13. Transfer collected fractions into prepared sample tubes. Keep fractions on ice during the day or store at -80°C until use.

3. **Bind-elute chromatography on HiScreen Capto Core 700 column**
3.1. Set up the chromatography system (Fig.4A), and equilibrate it at room temperature for 15 min. Degas all solutions.
3.2. Wash the column with 24mL PBS at 2mL/min.
3.3. Load 1.5-1.7mL sample from the pooled fractions (F6-7-8 from large-scale DGUC) on the column. The rate of application should be approximately 1mL/min. Wash sample into the column with 500µL PBS with a 1mL syringe.
3.4. Start perfusion with 2mL/min. Collect 12× 500µL fractions without stopping perfusion. If all the desired fractions are collected, stop the perfusion.
3.5. Optional: If the composition of captured proteins is the object of research interest, 0.1M NaOH in 30% 2-propanol wash can be performed, and cleaning-in-place fractions (CIPF) can be collected. Change the perfusion apparatus including stopcock and silicone tubing to another one. Perfuse 25mL 0.1M NaOH in 30% 2-propanol at 0.2mL/min, then collect 20× 500µL CIPFs. Add 500µL 0.1M HCl to collected CIPFs to neutralize fractions. NOTE: Neutralization should be performed after 2mL wash (CIPF5) to avoid low pH in first fractions.
3.6. For column regeneration, perfuse10mL 1M NaOH in 30% 2-propanolat 0.2mL/min.

3.7. Change the perfusion apparatus, and wash the column with 10-15mL PBS at rate 2mL/min. Check the pH of eluted solution, and continue wash until pH is 7.2-7.4.

3.8. If another isolation is performed, next sample can be loaded. If no other isolation is performed, perfuse 24mL 20% ethanol in PBS, at 2mL/min. Close the column, and store it at 4°C.

4. Size-exclusion chromatography on ExoSpin™ gravity-eluted column

4.1. Set up the chromatography system (Fig.4B), and equilibrate at room temperature for 15 min. Prepare 1.5mL sample tubes for fraction (ES) collection. Degas all solutions.

4.2. Discharge storage buffer from the top of the column.

4.3. Wash the column with 2×10mL PBS using gravity.

4.4. Load 500µL from pooled fractions (F5-7 from small-scale DGUC) onto the column by spreading sample on the top of the resin by pipetting it slowly. Collect the first 500µL fraction.

4.5. Load the second 500µL sample, then collect the second fraction.

4.6. Start washing the column by loading 500µL PBS, and collect fractions in an empty sample tube. Repeat step for 20-30 times.

4.7. ES fractions should be kept on ice until use or store them at -80°C for longer time.

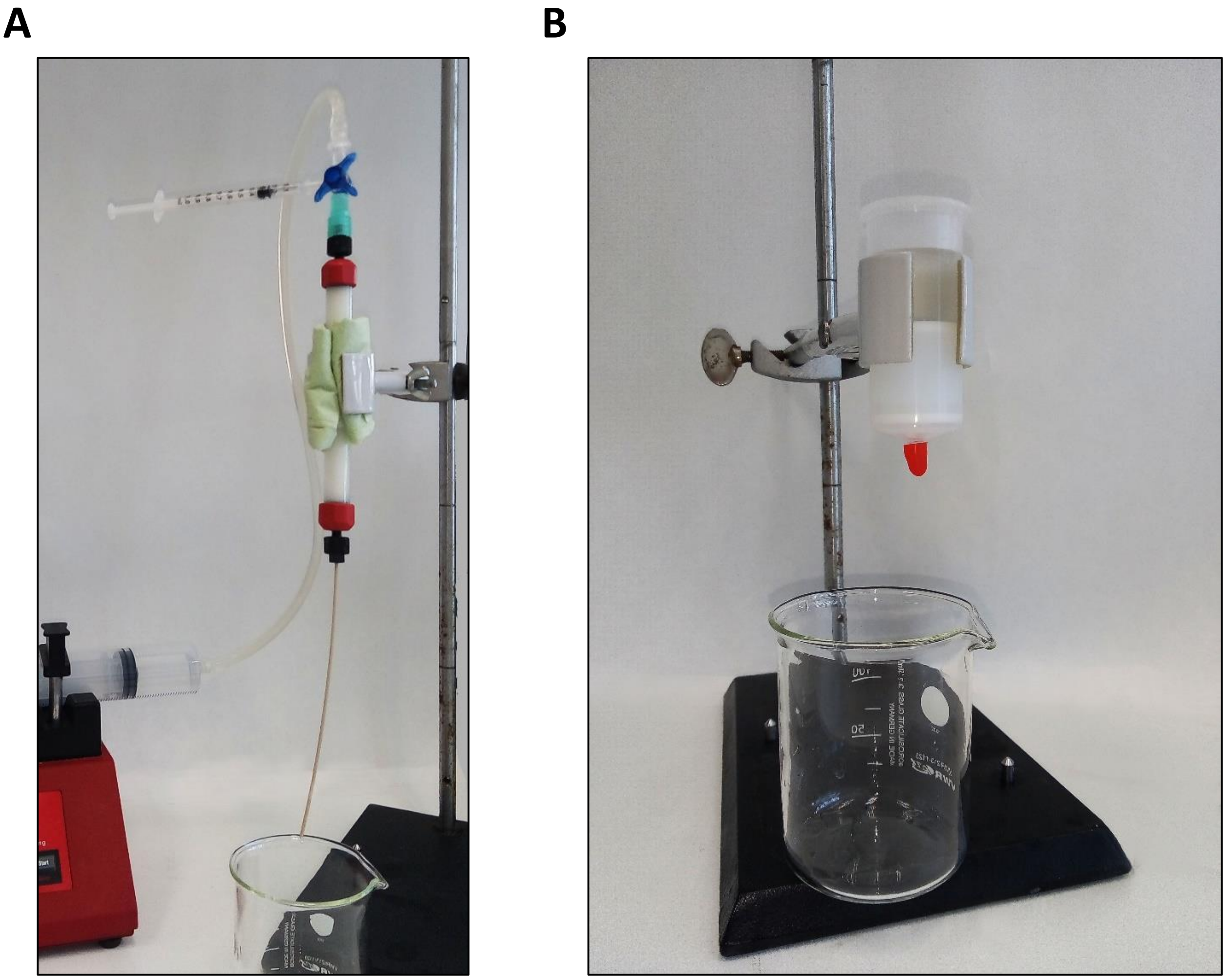

Figure 4 – Set up of chromatography system for HiScreen Capto Core 700 (A) and Exo-Spin™ (B) columns
